# Impact of Clear Aligners on the Oral Microbiome: A Systematic Review of Current Evidence

**DOI:** 10.1002/cre2.70265

**Published:** 2026-03-18

**Authors:** Mariangela Tabone, Carmen Martin Carreras‐Presas, Niccoló Cenzato, Massimo Del Fabbro, Rocio Gonzalez Soltero

**Affiliations:** ^1^ Faculty of Biomedical and Health Sciences Universidad Europea de Madrid Madrid Spain; ^2^ Department of Dentistry, Faculty of Biomedical and Health Sciences Universidad Europea de Madrid Madrid Spain; ^3^ Department of Biomedical, Surgical and Dental Sciences, School of Dentistry University of Milan Milan Italy

**Keywords:** biofilms, clear aligners, fixed aligners, oral malocclusion, oral microbiota

## Abstract

**Objectives:**

Oral malocclusion affects a large portion of the population, impacting both function and aesthetics. Recent advances in orthodontics offer a variety of treatment options, including traditional fixed appliances (FA) and, more recently, clear aligners (CA). This systematic review aims to synthesize and critically evaluate current evidence on oral microbiological changes during orthodontic treatment, with a focus on CA.

**Materials and Methods:**

A comprehensive literature search was conducted using PubMed, Medline, Cochrane Library, Embase, and Scopus. Keywords included: (“Bacteria” OR “Microbio*”) AND (“Orthodontic aligners” OR “Invisalign”). The review was registered in PROSPERO (CRD420240628072), and the final search was completed on October 15, 2024. Two reviewers independently screened studies, extracted data, and assessed risk of bias. Primary outcomes included changes in oropharyngeal microbiota (salivary, lingual, gingival, and pharyngeal) and functional diversity (e.g., alpha and beta diversity, metabolic pathways).

**Results:**

Twelve studies met inclusion criteria. Findings revealed that CA use leads to time‐dependent shifts in the oral microbiome, with notable changes in beta diversity within 24 h. Over periods from 1 to 12 months, CA users exhibited more favorable oral health outcomes compared to FA users, including lower plaque index (PI), reduced gingival inflammation (GI), and decreased probing pocket depth (PPD). These improvements are likely to be due to the easier hygiene maintenance of CA.

**Conclusion:**

Clear aligners appear to promote better oral microbiological balance and periodontal health than FA, likely due to easier hygiene maintenance and reduced plaque accumulation, with consistently lower levels of *Streptococcus mutans* observed in subjects undergoing CA therapy.

## 1 Introduction

Oral malocclusion is a widespread health issue, affecting between 39% and 93% of young adults, according to World Health Organization data, making it the third most significant oral health problem after periodontal disease and dental caries (Lone et al. 2024; Cenzato et al. 2021). This condition not only disrupts the function of the orofacial system but also has long‐term implications for patients' psychosocial well‐being and self‐esteem (Cenzato et al. 2021; Cassetta et al. 2020). Many etiological factors for malocclusion have been proposed, with genetic, environmental, and ethnic factors being the main contributors (Cassetta et al. 2020).

**Figure 1:**
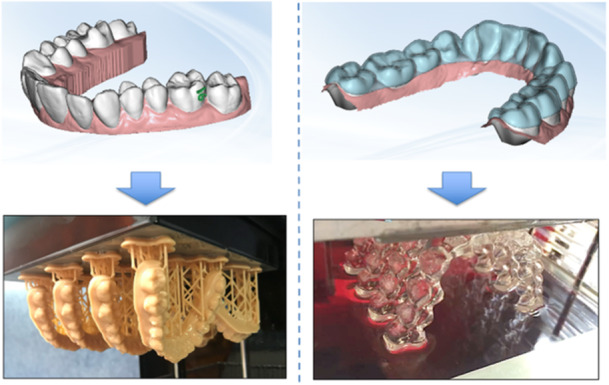
Production of aligners through thermoforming versus 3D‐printed aligners. Courtesy of Galuppo Laboratory.

To address this problem, orthodontic therapy aims to correct malocclusion and craniofacial skeletal discrepancies while enhancing oral functions such as mastication, deglutition, and breathing (Rouzi et al. 2023). This therapy involves applying controlled forces to the teeth through orthodontic appliances, which stimulate bone remodeling: bone resorbs on one side of the tooth and forms on the other, allowing for proper dental alignment (Li et al. 2018). Historically, fixed appliances (FA) were the predominant method used in orthodontics but recently the introduction of clear aligners (CA) has revolutionized the field (Cenzato, Occhipinti, et al. 2024; Bichu et al. 2023). Suitable for both adults and younger patients, these small, transparent thermoplastic devices offer a more discreet and comfortable alternative to conventional braces (Cenzato, Occhipinti, et al. 2024). Recent advancements in CAD‐CAM technology and transparent thermoplastic materials have further enhanced CA therapy, meeting the growing patient demand for more aesthetically pleasing and comfortable orthodontic solutions (Bichu et al. 2023). Additionally, removable aligners facilitate better oral hygiene by allowing patients to clean their teeth more effectively, reducing the negative effects typically associated with FA (Rouzi et al. 2023; Shokeen et al. 2022). Today, numerous aligner brands are available, differing primarily in material composition, wear time, gingival margin design, and the inclusion of attachments and accessories (Tartaglia et al. 2021). The latest thermoplastic materials have made CA more flexible, better adapted to the dental arch, and consistent in delivering orthodontic forces (Tartaglia et al. 2021). Through 3D scanning, clinicians can now create highly accurate digital models of dental arches. This technology allows for precise planning of tooth movements to realign teeth and correct malocclusions. The resulting digital models are used to produce photopolymer resin prints or thermoformed aligners, which patients wear sequentially for 18–20 h a day (dos Santos et al. 2012; Jindal et al. 2019) (Figure 1). As a result, the demand for orthodontic treatments has surged, with many patients increasingly preferring innovative aesthetic appliances over traditional options (Alansari et al. 2019). In addition to their aesthetic and comfort benefits, CA also offer significant oral health advantages. Compared to fixed orthodontic appliances, CA are associated with fewer complications, enhanced aesthetics, and improved patient hygiene, all of which contribute to better overall oral health (Cenzato et al. 2022). Specifically, CAs help reduce food deposits and dental plaque, which, if left unmanaged, can lead to gingival inflammation (GI) and bacterial proliferation (Cenzato et al. 2022; Zheng et al. 2024).

However, it is crucial to consider both the direct and indirect impacts of any orthodontic appliances on the oral environment, microbiome, and periodontal tissues (Zheng et al. 2024; Zhao et al. 2020). Oral health indexes, such as the decayed, missing, and filled teeth index and the periodontal index (PI), provide standardized methods for evaluating the prevalence and severity of dental caries and periodontal diseases, respectively (Moradi et al. 2019). These indices are essential for epidemiological studies and for planning public health interventions.

**Figure 2:**
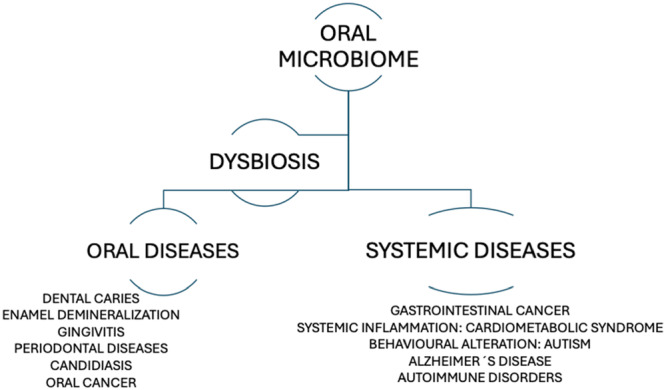
The figure summarizes some of the most interesting associations described in the literature regarding the influence of the oral microbiome and dysbiosis at both local and systemic levels.

The microbiome is defined as the set of microbial ecosystems populating the different niches of the organism. Many studies show there is a relationship between the microbiome and the different systems and organs of the human body, thus influencing our physiology (Gilbert et al. 2018; Bello‐Corral et al. 2023; Inchingolo et al. 2024). The oral microbiome plays a pivotal role in health and disease and could influence these parameters. Oral diseases such as periodontal disease and dental caries are prevalent worldwide. These conditions are often linked to dysbiosis in the oral microbiome, wherein disease‐related bacteria outnumber beneficial ones (Yama et al. 2023). The term dysbiosis or dysbacteriosis refers to an imbalance in the number or type of microbial colonies present in the body. It is closely associated with the most common oral diseases such as periodontal disease or dental caries (Figure 2). Recently, the oral microbiome has been suggested as a diagnostic and prognostic biomarker in head and neck cancer (Benjamin et al. 2023; Constantin et al. 2023). The presence of an overgrowth and overpopulation of Gram‐negative species such as *Prevotella, Treponema, Fusobacterium* and *Porphyromonas gingivalis* is associated with chronic inflammation and the progression from gingivitis to periodontitis in susceptible patients (Martínez‐García and Hernández‐Lemus 2021). In patients undergoing orthodontic treatment, white spots are usually precursors of dental caries. Orthodontic FA can make oral hygiene difficult, leading to plaque accumulation and cariogenic bacterial overgrowth (Cenzato, Occhipinti, et al. 2024; Carvalho et al. 2023). Oral dysbiosis may induce also dental caries. Veenman et al. (2024) conclude in a recent systematic review that some taxa such as *Prevotella denticola* (*P. denticola), Scardovia wiggsiae (S. wiggsiae), Streptococcus sobrinus (S. sobrinus)*, and *Streptococcus mutans* (*S. mutans*) may be associated with increased caries risk. Moreover, it is well known that levels of cariogenic bacteria like *S. mutans* and *Lactobacilli* can increase, especially during FA treatment leading to a higher risk of dental caries (Maheshwari et al. 2024).

The implications of oral dysbiosis extend beyond the oral cavity, affecting various systems in the body. Oral dysbiosis has been associated with systemic diseases such as cardiometabolic syndrome (diabetes, hypertension, dyslipidemia or obesity) neurological alterations and Alzheimer´s disease, autoimmune disorders such as inflammatory bowel disease, rheumatoid arthritis, systemic lupus erythematosus, Sjogren syndrome, ankylosing spondylitis, multiple sclerosis or autoimmune liver diseases (Benjamin et al. 2023; Constantin et al. 2023; Garcia‐Gutierrez et al. 2024; Gabriel and Ferguson 2023; Bello‐Corral et al. 2023; Trouw et al. 2009; Huang et al. 2023). Lastly, head and neck cancer or gastrointestinal cancer have also been associated with oral dysbiosis (LaCourse et al. 2021; Evenepoel et al. 2024).

Interestingly, oral dysbiosis may also have behavioral implications, particularly in children with autism spectrum disorders. According to recent studies, the dysbiotic oral microbiome can potentially affect behavior through the blood‐brain‐barrier pathways, the gut‐brain‐axis pathways or the chronic inflammation of periodontal tissue leading to lipopolysaccharide leakage to the blood‐brain barrier (Sampaio‐Maia et al. 2016).

Moreover, it has been described that the placement of orthodontic appliances can qualitatively alter the structure of plaque biofilm and induce inflammation. Uncontrolled inflammation during orthodontic treatment is undesirable therefore, maintaining excellent oral hygiene throughout orthodontic treatment is essential to prevent deterioration of dental and periodontal health (Tortora et al. 2023).

Considering all, it seems clear that the management of oral microbiota and periodontal health is critical during orthodontic treatments. Understanding the interactions between the oral microbiome and host Wis is crucial for developing targeted therapies and preventive measures (Galluccio et al. 2021; Jiang et al. 2018; Alqerban 2021; Levac et al. 2010; Campobasso et al. 2021). While orthodontic treatment can significantly benefit dental alignment and overall oral health, it's important to be aware of its potential effects on the oral microbiome. Thus, the objective of this review is to critically evaluate the current evidence on oral microbiology during orthodontic treatment with CA. Addressing the potential associations between the specific microbial composition of the oral microbiome during CA treatment (*question 1*) and the impact of CA treatment on oral health parameters influencing the microbiome (*question 2*).

## 2 Materials and Methods

### 2.1 Protocol

The present review has been carried out in accordance with the PRISMA (Preferred Reporting Items for Systematic Review and Meta‐Analyses) guidelines and was registered in Prospero number 628072 (Moher et al. 2009). The PICO framework (Population, Intervention, Comparison, and Outcomes) was developed to answer the research question: In patients undergoing orthodontic treatment with CA, how does the use of CA affect changes in oropharyngeal microbiota, including salivary, lingual, gingival, and pharyngeal composition, clinical parameters such as PI, GI, bleeding on probing (BOP), pocket probing depth (PPD), as well as functional diversity (e.g., alpha and beta diversity, metabolic pathways), compared to baseline or other orthodontic treatments?

- Population (P): Patients undergoing orthodontic treatment with CA
- Intervention (I): Use of CA.
- Comparison (C): Not specified (could include comparisons to FA or untreated populations).
- Outcome (O): Changes in oropharyngeal microbiota, including salivary, lingual, gingival, and pharyngeal composition, clinical parameters such as PI, GI, BOP, PPD as well as functional diversity (e.g., α and β diversity, metabolic pathways).

Alpha and beta diversity metrics are used to characterize microbial community structure and assess the impact of various environmental or experimental factors. Alpha diversity measures species' richness and evenness within individual samples and is commonly used to infer ecosystem complexity or stability. Beta diversity is used to evaluate compositional differences between sample groups, revealing how microbial communities shift in response to external influences such as diet, antibiotic use, or environmental stressors. These metrics are used to identify dysbiosis by revealing a decrease in alpha diversity and/or shifts in beta diversity that could indicate changes in microbial composition associated with unhealthy states.

### 2.2 Search Strategy

A systematic literature search was conducted using Medline, Cochrane Library, Embase and Scopus databases, the keywords used were: (“Bacteria” OR “Microbio*”) AND (“Orthodontic clear aligners” OR “invisalign”). The same search equation was initially conducted on PubMed and subsequently expanded using the OvidSP software, available at the Universidad Europea de Madrid, which enables simultaneous searching across other specialized databases. No constraints on study duration or publication date were applied to ensure comprehensive literature coverage from inception to December 2024. Cross‐references of previous reviews and relevant articles were also scanned for additional studies.

### 2.3 Eligibility Criteria

The inclusion criteria were studies (1) involving patients with orthodontic treatment using CA, (2) published in English in peer‐reviewed journals, (3) reporting data on oropharyngeal microbiota composition (presence and relative abundance of taxa at the phylum [p], family [f], genus [g], and species [s] levels), as well as functional diversity, and (4) using interventional or observational study designs, including cross‐sectional, case‐control, and prospective and retrospective cohort studies. Preclinical studies or studies involving children, adolescents, or older adults (> 65 years old) were also included. For this review were not included Books, editorials, letters to the editors, corrigendum, literature reviews, meta‐analyses, case reports, clinical vignettes, proceedings, theoretical articles, qualitative studies, gray literature, as well as conference abstracts and newsletters.

No further restrictions were applied, such as publication year, geographic location, clinical setting, gender, or ethnicity of participants.

### 2.4 Bias Analysis

The Jadad scale (Clark et al. 1999) was initially applied for potential application to assess study quality (Table S1). This tool evaluates criteria such as randomization, double‐blinding, and the reporting of withdrawals, which are key for randomized controlled trials. As the studies under review were non‐randomized cross‐sectional designs, we secondly utilized the Appraisal Tool for Cross‐Sectional Studies (AXIS) to assess potential biases in cross‐sectional studies (Moher et al. 2009). The tool evaluates key aspects, including the clarity of objectives, study design appropriateness, sample size justification, data handling, and transparency in reporting. For non‐response or missing data, the presence or absence of mitigation strategies was documented. Additionally, we reviewed the statistical methods and ethical considerations reported in each study to ensure replicability and adherence to standards.

### 2.5 Study Selection

Two authors (N.C. and M.T) independently screened the titles and abstracts of the selected studies after removing duplicates to determine their relevance to the research questions. Full‐text evaluation was conducted for studies that appeared to meet the inclusion criteria or had incomplete information in their titles and abstracts. Studies failing to meet the eligibility criteria were excluded from the review. Additionally, the authors examined the references of identified articles for other relevant studies. In cases of disagreement regarding study inclusion, a third author (R.G.S.) was consulted, and conflicts were resolved through collaborative discussion.

### 2.6 Data Extraction and Presentation of Results

Data from the studies included were extracted by two independent reviewers (N.C. and M.T.). Extracted information included 12 research articles and their corresponding oral microbiota outcome data, and other relevant clinical results. Any disagreements that arose between the reviewers were resolved by the other two reviewers (C.M.‐P. and R.G.S.).

## 3 Results

**Figure 3:**
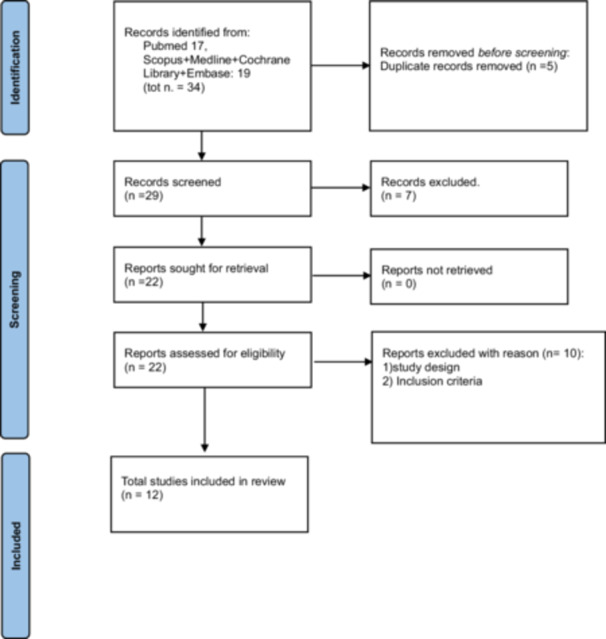
Flowchart representation.

A total of 34 studies were identified from all the databases used (16 in PubMed and 18 from the aggregation of the other aforementioned databases using the OvidSP software). Following the electronic search, all the identified records were collated and uploaded to the online software Covidence (https://www.covidence.org/), where the five duplicates were removed. The titles and abstracts of the remaining 29 were initially screened by two independent reviewers (N.C. and M.T.) for assessment against the inclusion and exclusion criteria, written above, and eliminating all those that were not strictly relevant. Specifically, of the seven articles excluded, it was clear from the abstract that four did not include data on oropharyngeal microbiota, and three were literature reviews. For those that were not clear enough to determine with certainty whether to include or exclude the article, we decided to read the article. Most of the discarded articles focused on biomechanical forces in the two different types of treatments or introduced specific dental hygiene products that could alter microbiological results. This step led to the selection of 12 studies (Figure 3). The full text of articles that met the inclusion criteria was then assessed in detail against the criteria. The inconsistencies in screening decisions were resolved through consultation with another reviewer (C. M.C.‐P.).

### 3.1 Descriptive Analysis of the Analyzed Studies

Twelve articles were included for qualitative synthesis. All the included studies were considered observational, cross‐sectional and longitudinal studies. The follow up period was from 4 h to 12 months.

Concerning the approach to study the changes in the bacterial composition, the analyzed studies employed three distinct methodologies. First, one study used quantitative PCR (qPCR) for specific bacteria and two others the CRT bacteria system (Ivoclar Vivadent, Schaan, Liechtenstein) also to quantify specific bacterial taxa. The remaining 9 studies follow a metagenomic approximation studying different regions for the 16S rRNA gene sequencing targeting the V3‐V4 (8 studies) and V1‐V3 regions (HOMING protocol), 1 study. The results are summarized in Tables 1 and 2 while a complete table (Supporting Information Table) is available in the Supporting Information.

**Table 1:** Summary of studies on microbiome and CA and their outcomes.

Article No.	First author and publication year	Title	Country	Type of study and objective	Sample size and sex
1	Sifakakis et al. (2018)	Salivary levels of cariogenic bacterial species during orthodontic treatment with thermoplastic aligners or fixed appliances: a prospective cohort study	Greece	Prospective, cohort study. Assessment of cariogenic bacteria *Streptococcus mutans, Lactobacillus acidophilus,* and *Streptococcus sanguinis*	30 adolescents (17 female and 13 males).
2	Mummolo, Nota, et al. (2020)	Salivary levels of *S. mutans* and Lactobacilli and other salivary indices in patients wearing clear aligners versus fixed orthodontic appliances: An observational study	Italy	Observational study. Investigating salivary levels of *S. mutans* and *Lactobacilli* and salivary parameters.	80 adults patients (34 female and 46 males)
3	Zhao et al. (2020)	The dynamics of the oral microbiome and oral health among patients receiving clear aligner orthodontic treatment	China	Prospective study. To monitor the impact of Invisalign appliances on the oral bacterial community and oral health	25 adult patients (22 female and 3 males)
4	Duong et al.(2022)	Salivary and plaque microbiomes during treatment with different orthodontic appliances.	USA	Observational and prospective study (4 times), comparing oral hygiene, PI and GI, microbiome in different study groups	20 adult patients (13 female, and 10 males)
5	Mummolo, Nota et al. (2020)	Salivary concentrations of *S. mutans* and Lactobacilli during an orthodontic treatment. An observational study comparing fixed and removable orthodontic appliances.	Italy	An observational and prospective study, comparing oral hygiene, plaque presence, and microbiome in different study groups CA group; MB group; RP group	90 adult patients: (34 female and 56 males)
6	Song et al. (2023)	Microbiome and metabolome associated with white spot lesions in patients treated with clear aligners	China	Cross‐sectional study. To investigate the differences in the salivary microbiome and metabolome between adolescents with and without White Spot Lesions (WSLs) treated CA.	205 adolescents: (sex: NA)
7	Wang et al. (2023)	Salivary microbiomes vary among orthodontic appliances and associate with clinical periodontal parameters.	China	Observational and prospective study (3 times), characterize and compare salivary microbiome between FA and CA and correlate with periodontal parameters	21 adults: (18 female and 3 males)
8	Yan et al. (2021)	Changes in the microbiome of the inner surface of clear aligners after different usage periods	China	Longitudinal study. To observe changes in the microbial composition on the inner surface of CA over different usage periods (1, 2, 3, 4 weeks of use)	8 adults: (8 female and 0 males)
9	Shokeen et al. (2022)	The impact of fixed orthodontic appliances and clear aligners on the oral microbiome and the association with clinical parameters: A longitudinal comparative study	USA	Longitudinal study. To provide a comprehensive, longitudinal view of the microbiome for orthodontic treatment with FAs compared with CAs.	24 participants (8–56 years): (16 female and 8 males)
10	Rouzi et al. (2023)	Characteristics of oral microbiota and oral health in the patients treated with clear aligners: a prospective study	China	Prospective study. To monitor the impact of CA on oral hygiene and periodontal health	15 adults: (9 female and 6 males)
11	Zheng et al. (2024)	Comparative characterization of supragingival plaque microbiomes in malocclusion adult female patients undergoing orthodontic treatment with removable aligners or fixed appliances: A descriptive cross‐sectional study.	China	Cross‐sectional study. To investigate the effect of CA and FA on supragingival bacterial communities in adult female undergoing orthodontic treatment.	48 adults: (48 female and 0 males)
12	Wang et al. (2019)	Alterations of the oral microbiome in patients treated with the Invisalign system or with fixed appliances	China	Observational study. To investigate changes in the oral microbiome in patients treated with Invisalign (CA) or with FA.	26 adults: (Sex: NA)

**Table 2:** Type of bacteria examined measurement techniques, outcomes, and oral impact.

First author and publication year	Type of bacteria analyzed	Measurements techniques	Outcomes	Clinical impact
Iosif Sifakakis et al. (2018)	*Streptococcus mutans; L. acidophilus; Streptococcus sanguinis*	qPCR	*S. mutans* and *L. acidophilus* levels were similar in adolescents using thermoplastic aligners or self‐ligating appliances, but *S. sanguinis* was lower with aligners.	Significant differences in plaque (s‐PlI) and gingivitis (s‐GI) scores favored thermoplastic aligners over fixed appliances. Male patients had higher plaque scores than females. Pre‐treatment oral hygiene correlated with plaque and gingivitis during treatment. No clear pattern of oral hygiene change over time was observed
Mummolo, Nota, et al. (2020)	*S. mutans, Lactobacilli*	CRT prevention system (Ivoclar Vivadent Clinical, Schaan, Liechtenstein).	Clear aligners (CA) support better oral hygiene than metal braces (MB). After 6 months, only 8% of CA users had high *S. mutans* levels compared to 40% of MB users. Neither CA nor MB affects salivary flow or buffering power	At t0, the CA group had significantly lower salivary flow compared to the MB group (*p* = 0.01). Saliva buffering power remained stable over time for both groups. After 6 months, only 8% of CA participants had high‐risk microbiota levels and maintained better oral hygiene than MB participants. About 40% of MB participants had increased caries risk and needed additional plaque control, while CA users faced less than 10% risk
Zhao et al. (2020)	Whole microbiota analysis	16S rRNA	No significant differences in alpha or beta diversity between before and after Invisalign treatment. After treatment, patients showed increased *Bacillus* and decreased *Prevotella* abundance.	After 6 months of Invisalign, plaque index (PI) scores significantly decreased, while bleeding on probing (BOP) and pocket probing depth (PPD) remained unchanged. Brushing frequency increased, indicating better oral hygiene. No significant correlations were found between microorganism abundance and clinical parameters
Duong et al. (2022)	Whole microbiota analysis	16S rRNA	No α‐diversity changes. β diversity: saliva had more *Streptococcus*, plaque had more *Actinomyces*. Fixed aligners (FA) group saw increased *Veillonella* and decreased *Haemophilu*s; clear aligner (CA) group showed variable Haemophilus, no *Veillonella* change	Clinical parameters significantly worsened during orthodontic treatment with fixed appliances (FA), but remained stable with clear aligners (CA). The microbial composition of plaque and saliva varied significantly between the two treatment groups. Despite these differences, changes in the abundance of certain genera were similar in both saliva and plaque, correlating with the associated clinical parameters. These genera may serve as potential biomarkers for health and disease.
Mummolo, Nota, et al. (2020)	*S. mutans, Lactobacillus*.	CRT bacteria (Ivoclar Vivadent Clinical, Schaan, Liechtenstein).	After 6 months, about 40% of fixed orthodontics (MB) patients had high‐risk levels of *Streptococcus mutans* and *Lactobacilli*, compared to 10% of Clear aligners (CA) and 13.3% of removable positions (RP) patients. MB patients had odds ratios of 5.05 and 4.33 for high‐risk *S. mutans* and *Lactobacilli*, respectively, compared to (CA) and (RP) patients.	Clear aligner (CA) patients maintained a plaque index of 0, while multi‐bracket (MB) patients had a significant increase in plaque over time
Song et al. (2023)	Whole microbiota and metabolome analysis	16S rRNA	α‐diversity is lower in white spot lesions (WSL) groups. β‐diversity shows some separation between WSL and control groups. Both groups share predominant phyla: *Firmicutes, Proteobacteria, Bacteroidetes, Actinobacteria, Fusobacteria, and TM7*. Common genera include *Streptococcus, Veillonella*, and *Neisseria*. WSLs have 14 taxa with higher abundance, such as *Actinobacteria* and *Rothia*, while 58 taxa, including *Firmicutes* and *Cetobacterium*, are enriched in controls.	Salivary microbial and metabolomic data correlated, showing clear aligners' impact on oral flora metabolism. Increased *Lachnoanaerobaculum, Rothia, Subdoligranulum*, and certain amino acids predicted WSL development
Wang et al. (2023)	Whole microbiota analysis	16S rRNA	α‐diversity was higher in fixed aligners (FA) than in clear aligners (CA). β‐diversity, including weighted‐Unifrac and Bray‐Curtis dissimilarities, was higher in CA. Four genera—*Selenomonas, Stomatobaculum, Olsenella*, and *Faecalicoccus*—were more abundant in FA (*p* < 0.05). Fifteen species, including *Prevotella salivae* and *Megasphaera micronuciformis*, were also more enriched in FA. FA creates distinct salivary microbial communities with higher richness, evenness, and different bacterial structures compared to CA	Plaque index (PI) increased during six months for fixed appliance (FA) patients but remained stable for clear aligner (CA) patients. Significant associations between PI, gingival index (GI), and probing depth (PD) were found in the FA group but not in the CA group. FA patients had more disease‐related bacteria, linked to worsening periodontal parameters
Yan et al. (2021)	Whole microbiota analysis	16S rRNA	α‐diversity decreases with aligner use, while β‐diversity slightly increases from T0 to T24. At the phylum level, *Firmicutes* increased by 24 weeks, whereas *Actinobacteria* and *Proteobacteria* were higher at 0 weeks. At the order level, *Lactobacillales* and *Bacteroidales* were high at 12 and 24 weeks, and *Actinomycetales* decreased from 0 weeks. At the genus level, *Streptococcus, Haemophilus*, and *Porphyromonas* increased, while *Rothia*, *Lautropia*, and Actinomyces decreased. At the species level, *Streptococcus infanti*s increased at 24 weeks, and *Rothia dentocariosa* and *Streptococcus anginosus* increased at 8 weeks. *Rothia aeria*, a pathogen, increased at 0 weeks	The pH of the aligner liquid decreased with prolonged use, becoming significantly more acidic after 12 h, which promotes demineralization.
Shokeen et al. (2022)	Whole microbiota analysis	16S rRNA	β‐diversity differed between the two treatment groups, but alpha diversity did not. Genus‐level differences were generally not significant except for *Leptotrichia* in fixed aligners (FA). The anterior and posterior plaque in clear aligners (CA) showed similar profiles, but tray plaque had a distinct profile.	Plaque (PI) and gingival indexes (GI) increased significantly in the FA but not the CA group
Rouzi et al. (2023)	Whole microbiota analysis	16S rRNA	In aligner tray plaque, alpha diversity indices were significantly lower at T3 compared to T0.	A reduction in plaque index and an improvement in periodontal health were observed. In plaque samples from aligner trays, the relative abundance of *Streptococcus* increased significantly, while microbial richness and diversity decreased notably with the duration of treatment. In subgingival plaque samples, however, no significant changes were detected in the α or β‐diversity of the microbiota.
Zheng et al. (2024)	Whole microbiota analysis	16S rRNA	Significant difference in α and β‐diversity were observed among the groups. Traditional fixed bracket (group B) ‘s microbiome had a higher abundance of gram‐negative bacteria. At the phylum level, *Actinobacteriota* was notably more prevalent in control group (group C), while *Bacteroidota* was enriched in Group B. Family‐level analysis showed increased levels of *Saccharibacteria* (formerly TM7) and *Prevotellaceae* in Group B. At the genus level, *Lautropia* was significantly more abundant in Group AT.	Fixed orthodontic appliances increased the relative abundance of anaerobes and periodontal pathogens in the oral microbiome
Wang et al. (2019)	Whole microbiota and inference metagenome	16S rRNA	Both fixed and Invisalign orthodontic treatments caused oral microbiome dysbiosis. Significant differences were found in *Firmicutes* and TM7 at the phylum level, and *Neisseria* at the genus level. The Invisalign group showed a higher predisposition to periodontal diseases and was enriched in energy, amino acid, carbohydrate, and terpenoid metabolism. In contrast, the fixed appliance group had increased membrane transport and nucleotide metabolism.	Both fixed and Invisalign treatments caused oral microbiome dysbiosis. The Invisalign system did not perform better than fixed appliances in terms of microbiome composition and function, contradicting previous claims of its periodontal health benefits

To clarify the results, they have been divided according to the two secondary research questions we aimed to address:

*Q1. Potential associations between specific microbial composition of the oral microbiome during CA treatment.*

The evidence collected from the literature review reports on various studies investigating the effects of CA on different oral health parameters and oropharyngeal bacteria and microbiota over a range of time periods. These studies span from short‐term evaluations of 4–24 h to long‐term assessments extending up to 12 months. The main outcomes extracted from these studies are the following:

In the first 4 h, Zheng et al. found significant variances in alpha and beta diversity among the two groups: CA and FA. FAs were linked to oral microbiome changes, notably an enhanced relative abundance of anaerobes, including periodontal pathogens (Zheng et al. 2021).

Yang et al. (2021) examined the changes in the microbiome of the inner surface of CA after different usage periods along 24 h. The findings revealed time‐dependent shifts in the microbial communities on the inner surfaces of the aligners, indicating that the duration of aligner use affects during the first 24 h of usage (alpha diversity decrease and beta diversity slightly increased from 0 to 24 h). At phylum level, the abundance of Firmicutes was higher when using CA (Wang et al. 2023).

Sifakakis et al. (2018) investigated in a cross‐sectional study the salivary levels of cariogenic bacterial species in the saliva of patients undergoing orthodontic treatment with either CA or FA for a period of 1 month. No differences were found in the salivary levels of *S. mutans* and *L. acidophilus* between adolescent patients treated for 1 month with CA or FA. Lower salivary levels of *S. sanguinis* were found in patients treated with thermoplastic aligners (CA) compared to those treated with FA (Sifakakis et al. 2018).

The 3‐month follow‐up conducted by Rouzi et al. (2023) found that in aligner tray plaque, alpha diversity indices were significantly lower over time. A reduction in plaque index and an improvement in periodontal health were observed. In plaque samples from aligner trays, the relative abundance of *Streptococcus* increased significantly, while microbial richness and diversity decreased notably with the duration of treatment. In subgingival plaque samples, however, no significant changes were detected in the α‐ or β‐diversity of the microbiota. Mummolo, Nota, et al. 2020 compared the salivary levels of *S. mutans* and *Lactobacilli spp*., along with other clinical parameters such as plaque index, buffering power of saliva and stimulated salivary flow, in patients wearing CA versus FA for 6 months (Mummolo, Nota, et al. 2020). CA significantly reduced the risk of developing dental caries, with only about 10% of CA patients showing high‐risk levels of *S. mutans* after 6 months, compared to approximately 40% of FA (multi‐bracket; MB) patients (Mummolo, Nota, et al. 2020; Mummolo, Tieri, et al. 2020). For the salivary flow, a statistically significant difference was found between the two groups at t0, with the CA group showing a low level of salivary flow (0 < mL/min < 1.5) more frequently than the MB group (*p* = 0.01). Regarding the buffering power of saliva, it remained stable over time in both groups, preventing the colonization of pathogenic microorganisms in the mouth and neutralizing acids produced by acidogenic bacteria, and enamel demineralization.

Zhao et al. (2020) conducted an observational study to examine the dynamics of the oral microbiome and oral health among patients receiving CA for 6 months. The researchers did not find significant changes in the alpha diversity (richness or diversity) before and after aligners treatment (Zhao et al. 2021). Wang et al. 2023 investigated the variations in salivary microbiomes among patients using different orthodontic appliances and their association with clinical periodontal parameters for 6 months comparing FA vs CA. Alpha diversity resulted higher for FA and beta diversity was higher for CA (Wang et al. 2023).

Beta diversity, both Weighted‐Unifrac and Bray‐Curtis dissimilarities, were statistically higher in CA. 4 genera: *Selenomonas, Stomatobaculum, Olsenella*, and *Faecalicoccus* were statistically higher (*p* < 0.05) in FA (Wang et al. 2023). At the species level, 15 species (*Prevotella_salivae, Megasphaera_micronuciformis, Selenomonas_sputigena, Dialister_ invisus, Olsenella_profus, Sutterella parvirubra, Prevotella_buccae, Cryptobacterium_curtum, Coprococcus_eutactus, S._wiggsiae, Veillonella_magna, Clostridium_ramosum, Lactobacillus_apis, Clostridium_spiroforme, Bacillus_thermocloacae*) were more enriched in the FA group versus the CA group. FA triggers salivary microbial communities distinct from CA, which are characterized by higher richness and evenness, distinct global bacterial community structures, higher bacterial correlations, and different bacterial taxa distributions. In the clinical parameters, PI showed an increasing trend during the 6‐month treatment in patients wearing FA but remained stable in patients wearing CA (Kaklamanos et al. 2023).

Twelve months follow up evaluations have been reported by Shokeen et al. (2022). Duong et al. (2016) and Song et al. (2023) explored the salivary and plaque microbiomes in patients undergoing treatment with different types of orthodontic appliances for 12 months.

Song et al. found that, when treated with CA, alpha diversity is lower in white spot lesions groups (WSL) and beta diversity (measured by unweighted UniFrac and Bray‐Curtis distance) showed a certain trend of separation between the two groups. At the phylum level, the predominant phyla in both groups were Firmicutes, Proteobacteria, Bacteroidetes, Actinobacteria, Fusobacteria, and Candidate division TM7. At the genus level, the most abundant genera (> 1% relative abundance) included *Streptococcus, Veillonella, Neisseria, Prevotella, Haemophilus, Porphyromonas, Actinomyces, Fusobacterium*, and *Rothia*. The analysis results (Lefse) revealed distinct taxa in the oral microbiome of individuals with WSLs and those without WSLs (Song et al. 2023). There were 14 taxa with higher abundances in the WSL group than in the control group, including Actinobacteria (at both the phylum and class levels). In contrast, 58 taxa, including Firmicutes, *Cetobacterium* and *Burkholderia*, were significantly enriched in the control group. There was a correlation between the salivary microbial and metabolomic datasets, reflecting the impact of CA on the metabolic activity of the oral flora and a concordant increase in the levels of *Lachnoanaerobaculum, Rothia*.

Shokeen et al. (2022), reported that beta but not alpha diversities were different between the 2 treatments groups. *Leptotrichia* resulted significantly different in FA but not in CA (Kaklamanos et al. 2023). PI and gingival indexes increased significantly in the FA but not the CA group.

Duong et al. (2016) found no changes in alfa diversity between groups. Differences in beta diversity analysis for saliva and plaque was observed for both the FA and CA groups. Saliva harbored a higher relative abundance of *Streptococcus* whereas plaque samples contained a greater abundance of Actinomyces in both FA and CA groups (Duong et al. 2016). FA group disease‐associated genus *Veillonella* increased in both plaque and saliva, whereas in the CA group, no change was observed (Zhao et al. 2021). The health‐associated genus Haemophilus decreased in both saliva and plaque of the FA group, while in the CA group, relative abundance varied but no consistent trend was observed.

*Q2. Impact of CA on oral health parameters impacting on the microbiome during clear orthodontic treatment.*

Aligners can cause changes in the oral microbiome which is defined as the group of more than 700 species that commonly coexist in the oral environment. CA generally help maintain better oral health compared to FA. This is primarily due to their removability, which allows for better oral hygiene practices.

Yang et al. (2021) found that the pH of the aligner liquid decreased as the duration of aligner usage increased in the first 24 h. The acidity of the aligner liquid contents increased significantly after 12 h of prolonged use. This result indicates that usage of aligners for 12 h or more creates an adverse trend for an acid‐induced demineralization environment.

Sifakakis et al. (2018), described a statistically significant difference in both plaque scores (s‐PlI) and gingivitis scores (s‐GI) was found between FA and thermoplastic aligners, which favored the latter. Oral hygiene was significantly associated with patient sex, with male patients having significantly higher plaque scores than female patients. Pre‐treatment oral hygiene levels were significantly associated with plaque scores and gingivitis during treatment.

Mummolo et al. described from a clinical point of view that only about 8% of CA participants achieve risky values of microbiota colonies after 6 months of treatment, with a stable plaque control, differently from MB participants. The maintenance of a better macroscopically PIand microscopically (*S. mutans* and *Lactobacilli* CFU) oral hygiene levels in CA participants, compared with MB participants. While in about 40% of MB participants there is an increased risk of dental caries and demineralization and so additional strategies for plaque control must be applied after the first 6 months of treatment (and for 20% just after 3 months), the use of CA seems to significantly limit this risk to less than 10% of the subjects. In their second study from these authors, patients wearing CA maintained a PI at level 0 throughout the study period (6 months). Patients with MB appliances experienced a statistically significant increase in the PI over time, highlighting the importance of plaque control and oral hygiene maintenance during orthodontic treatment (Wang et al. 2023).

Zhao et al. (2021) examined that after 6‐month of Invisalign treatment, the PI scores of patients significantly decreased, while BOP and PPD did not change. The brushing frequency of patients was significantly increased after treatment, and the number of times which together indicated that patients had better oral hygiene habits that corresponded with the changes in PI scores. Pearson correlation coefficients were calculated between the genera abundance and clinical data to investigate the association between oral microorganisms and the clinical parameters.

Wang et al. (2023) doing a follow‐up by 6‐months, identified significant associations between PI, GI and PD were observed in the FA group but not the CA group. Bacterial structures, including higher richness and evenness and higher abundance of disease‐related genera and species in the salivary bacterial communities harbored by patients wearing FA are associated with increasing periodontal parameters (PD, PI and GI). The appliance‐varied bacterial communities highlight the importance of developing appliance–orientated periodontal strategies during orthodontic treatments, which need further clinical trials (Kaklamanos et al. 2023).

For a 12‐months follow‐up, Shokeen et al. (2022) found that PI and GI increased significantly in the FA but not the CA group.

### 3.2 Risk of Bias

When analyzing all studies following Jadad scale for bias assessment, most of items received a score of 0, reflecting the lack of criteria such as randomization and double‐blind in their methodology (Supporting Information). Then the potential biases across the studies were evaluated using the AXIS scale for cross‐sectional studies. The results of this scale showed that all the analyzed studies defined their aims and objectives, reducing the likelihood of bias from unclear research goals (Table 3). The study design was appropriated for the aims in all cases, which suggests minimal bias in terms of methodological fit. However, potential bias arose regarding sample size justification. For instance, studies like Sifakakis et al. (2018), Mummolo, Nota, et al. (2020), and Zhao et al. (2020) did not provide adequate justification for their sample sizes. Additionally, some studies failed to adequately address non‐responders or missing data. Rouzi et al. (2023) applied regression imputation to missing data, while others either lacked this information or had no response rate data, which could be considered a source of bias if non‐responders were not properly accounted for. Regression imputation methods reduce potential bias from missing data. In contrast, many studies simply marked “N/A” for non‐responders, providing no clear information on how missing data was managed, which could leave room for bias due to unaccounted‐for non‐response. All studies justified their conclusions with the results, which helps mitigate bias. Furthermore, each study acknowledged its limitations, reducing the potential for over‐interpretation of findings. One key exception is Zheng et al. (2024), where the presence of conflicts of interest may have introduced bias, as financial or personal interests could have influenced the study's interpretation. Importantly, all studies secured ethical approval and consent informed where it was request.

**Table 3:** Potential bias evaluation of the included studies based on the AXIS assessment criteria.

	Article	Potential bias evaluation
1	Sifakakis et al. (2018)	Study following the patients 1 month (in this RCT plaque and bleeding index doesn't change when the time is longer when comparing CA and FA).
2	Mummolo, Nota, et al. (2020)	Data were analyzed by operators who did not know the origin group of the collected data. SELECTION BIAS related with the possible higher socioeconomic status of subjects treated with CA rather than MB considering that there was a reported association between oral hygiene level and socioeconomic status
3	Zhao et al. (2020)	Sex bias (22 female vs. 3 male).
4	Duong et al. (2022)	Absence of control group
5	Mummolo, Nota, et al. (2020)	Absence of control group. To avoid bias, the data were analyzed by operators who were blind to the fact that each collected data belongs to which group.
6	Song et al (2023)	White spot lesions (WSL) vs non‐WSL. They consider the control de NON‐ WSL.
7	Wang et al. (2023)	No control group
8	Yan et al. (2021)	No control group
9	Shokeen et al. (2022)	No control group
10	Rouzi et al. (2023)	No control group
11	Zheng et al. (2024)	Only female patients
12	Wang et al. (2019)	No clinical data as PI

## 4 Discussion

CA have gained popularity in orthodontics due to their aesthetic and practical advantages. This discussion synthesizes findings from 12 observational (cross‐sectional and longitudinal) studies on the impact of CA on the oral microbiome and oral health parameters in X cases compared to FA. The review found that CA facilitated better oral hygiene compared to self‐ligating and MB or FA.

From our analysis we can extrapolate three main outcomes to be considered in further detail:

### 4.1 Short‐Term Microbiome Effects of CA

Initial studies (Zheng et al. [2021]; Yang et al. [2021]) demonstrate that within 24 h of CA use, the oral microbiome undergoes significant shifts, characterized by a reduction in *alpha diversity* (microbial richness) and an increase in *beta diversity* (community composition variability). These shifts include an increased abundance of Firmicutes and anaerobic species, some linked to periodontal disease (e.g., *Porphyromonas*, *Fusobacterium*). Such early dysbiosis could predispose to inflammation and enamel demineralization, especially if oral hygiene is suboptimal or aligners are worn continuously without proper cleaning. This underscores the importance of early patient education on aligner maintenance to prevent microbial imbalances.

Medium‐term microbiological and clinical impacts (up to 6 months): Over a 6‐month period, studies such as those by Mummolo et al. and Wang et al. (2023) indicate that CA generally promote better oral health outcomes compared to FA. CA users experienced a significant reduction in the risk of dental caries, with only 10% of users showing high‐risk levels of *S. mutans* after 6 months, compared to 40% of FA users. Over a medium‐term period of up to 6 months, CAs have demonstrated a positive impact on reducing dysbiosis‐related risks compared to FA.

Long‐term changes in microbiota and oral health (12 months): Longer‐term studies, including those by Duong et al. (2016) and Song et al. (2023) explored microbiome dynamics and oral health outcomes over a 12‐month period. These studies confirmed that alpha diversity was generally lower in patients with WSLs, while beta diversity distinguished patients with and without WSLs. The CA group demonstrated more stable microbiota and periodontal health, with reduced risks of developing harmful conditions, while FA users exhibited an increase in disease‐associated genera, such as *Veillonella*, and higher PI and GI scores. The prolonged use of CA thus appears to limit the adverse impacts on the microbiome, promoting better overall oral health compared to FA over the course of 1 year. Prolonged CA use to promote periodontal stability and lowering the long‐term risks associated with dysbiosis and inflammatory oral conditions. This aligns with better overall clinical outcomes, suggesting that CAs may be a preferable option for patients concerned with maintaining oral health over extended treatment periods.

Studies also indicate that patients using CA tend to have better plaque control, lower PI, and reduced GI, leading to improved periodontal health over time (Rouzi et al. 2023). Other studies have shown a gradual decline in PI and PD during CA treatment, indicating improved periodontal health (Zhang et al. 2021; Yan et al. 2020; Al‐Moghrabi et al. 2020; Rossoni et al. 2020; Kim et al. 2020; Cenzato, Di Iasio, et al. 2024).

### 4.2 Dysbiosis During Orthodontic Treatment

Alpha and beta diversity metrics serve as essential indicators of dysbiosis (Table 2). Studies such as Zheng et al. (2024) and Yan et al. (2021) demonstrated that significant microbial shifts can occur as early as the first 4–24 h of CA use, marked by a decrease in alpha diversity and modest increases in beta diversity, indicative of reduced microbial richness and the beginning of community divergence. These early changes align with current evidence suggesting that enclosed anaerobic conditions under aligners create microenvironments that selectively favor certain bacterial taxa, particularly acidogenic and anaerobic species (e.g., *Prevotella*, *Fusobacterium*) (Zhao et al. 2020; Song et al. 2023). Over longer periods, such as one to 6 months, several studies (e.g., Rouzi et al. 2023; Mummolo, Nota, et al. 2020) observed a sustained decrease in alpha diversity on aligner surfaces and within salivary samples, which may reflect the onset of dysbiotic conditions, although often less pronounced than those observed with FA. Interestingly, while FA users tend to exhibit higher microbial richness and diversity (alpha diversity), this is not necessarily indicative of a healthy microbiome, as studies like Wang et al. (2023) and Haug et al. (2024) reported enrichment of disease‐associated genera such as *Veillonella*, *Selenomonas*, and *Scardovia* in FA‐treated patients. In contrast, CA users displayed more stable microbial communities over time, with fewer pathogenic shifts and better clinical outcomes (e.g., plaque index and gingival scores), potentially due to improved hygiene access. This supports the findings from Zhao et al. 2020 and Mummolo et al. indicating that CA treatment may mitigate the risk of caries and periodontal dysbiosis. A systematic review has also explored the impact of removable and fixed orthodontic appliances on the oral microbiome, confirming the growing scientific interest in this area (Lucchese et al. 2021). It reported that microbial changes were mostly transient and primarily focused on removable devices rather than specifically on CA (Lucchese et al. 2021). In further confirmation that the topic remains highly relevant, a recently published review directly compared FA and CA (Lucchese et al. 2025), highlighting that CA, which are the main focus of the present study, are generally associated with better microbiological and periodontal outcomes (Lucchese et al. 2025). Collectively, current evidence suggests that while both appliance types influence the oral microbiome, CA exerts a more controlled and transient impact, with dysbiosis largely dependent on oral hygiene behaviors and duration of appliance wear.

### 4.3 Clinical Impact of Clear Aligners on Oral Health

Patients using aligners had significantly lower PI and GI, and fewer patients showed high concentrations of *S. mutans*. CA users maintained stable periodontal parameters over time, while FA users showed an increasing trend in PI, GI, and probing depth (PD) (Zheng et al. 2021; Al‐Moghrabi et al. 2020; Rossoni et al. 2020). Additionally, CA significantly reduced the risk of developing dental caries, with only about 10% of CA patients showing high‐risk levels of *S. mutans* after 6 months, compared to approximately 40% of MB patients.

Additionally, CA significantly reduced the risk of developing dental caries, with only 8% of CA patients showing high‐risk levels of *S. mutans* after 6 months, compared to approximately 40% of MB patients (Mummolo, Nota, et al. 2020, Mummolo, Tieri, et al. 2020; Cenzato, Di Iasio, et al. 2024). Research also indicates that CAs can induce short‐term changes in the oral microbiome, which tend to stabilize over time, primarily affecting beta diversity. Some consensus is observed about maintained stable periodontal parameters over time in CA, while FA users exhibited increasing PI, GI, and PD. This suggests that CAs might be more effective in maintaining oral hygiene and preventing periodontal disease than FA Improvements in oral health parameters, such as PI and GI, suggest that CAs are a viable alternative to FAs, especially for patients who prioritize aesthetics and comfort. However, we may notice that prolonged CA use can increase oral acidity, potentially raising the risk of acid‐induced demineralization, WSLs, and caries, so associated risks underscore the need for vigilant oral care practices (Cenzato, Di Iasio, et al. 2024; Levrini et al. 2021). Compared with previous systematic reviews on similar topics, the present study expands the available evidence by including more recent clinical and microbiological studies, some with longer follow‐up periods. Moreover, this review integrates microbial and clinical parameters in a unified analysis, offering a more comprehensive perspective on the oral health implications of CA therapy.

Clinical outcomes, such as cariogenic bacteria levels and caries risk, appear influenced by CA use, though findings are mixed. Despite these insights, significant gaps remain in understanding the clinical implications of microbiome changes due to CA use and the long‐term health outcomes associated with these microbial shifts.

### 4.4 Limitation

This study has certain limitations due to the lack of a standardized methodology in the studies examined. Additionally, orthodontic treatment can have both positive and negative effects on oral hygiene, a crucial factor in fully understanding its impact on the microbiota. However, few studies have explored this correlation so far further research and specifically clinical trials would be necessary to accurately assess the impact of CA on changes in the oral microbiota.

## 5 Conclusion

Clear aligners appear to promote a healthier oral environment compared with FA. Short‐term changes in the microbiome indicate temporary shifts in diversity, emphasizing the need for proper hygiene and patient education. Over medium and long periods, clear aligners are associated with lower levels of *S. mutans*, reduced plaque and GI, and a more stable microbial community. Overall, prolonged use of CA supports better periodontal stability and lowers the risk of dysbiosis and related oral diseases. It would be valuable to conduct longitudinal and targeted investigations assessing microbiome dynamics both over the long term and at intraday intervals.

## Author Contributions

Conceptualization: Niccoló Cenzato, Mariangela Tabone, and Rocio Gonzalez Soltero. Methodology: Niccoló Cenzato. Software: Carmen Martin Carreras‐Presas. Validation: Niccoló Cenzato and Mariangela Tabone. Formal analysis: Mariangela Tabone. Investigation: Rocio Gonzalez Soltero and Carmen Martin Carreras‐Presas. Resources: Niccoló Cenzato and Rocio Gonzalez Soltero. Data curation: Mariangela Tabone and Rocio Gonzalez Soltero. Writing – original draft preparation: Niccoló Cenzato and Rocio Gonzalez Soltero. Writing – review and editing: Niccoló Cenzato and Rocio Gonzalez Soltero. Visualization: Mariangela Tabone and Niccoló Cenzato. Supervision: Niccoló Cenzato, Carmen Martin Carreras‐Presas, and Rocio Gonzalez Soltero. Project administration: Niccoló Cenzato and Rocio Gonzalez Soltero. All authors have read and agreed to the published version of the manuscript.

## Funding

The authors received no specific funding for this work.

## Consent

The authors have nothing to report.

## Conflicts of Interest

The authors declare no conflicts of interest.

## Supporting information

supplementary table.

## Data Availability

The data presented in this study are available in the Tables and more information can be requested from the corresponding author.
